# Comprehensive effect of Naoxintong capsule combined with Western medicine on coronary heart disease after percutaneous coronary intervention: a meta-analysis

**DOI:** 10.3389/fphar.2024.1274000

**Published:** 2024-03-25

**Authors:** Liyuan Yu, Peiying Huang, Meida Wang, Zhishang Li, Hairong Cai, Yuchao Feng, Lulu Wu, Weihang Peng, Jing Zeng, Bojun Chen

**Affiliations:** ^1^ The Second Clinical Medical School of Guangzhou University of Chinese Medicine, Guangzhou, China; ^2^ Guangdong Provincial Key Laboratory of Research on Emergency in Traditional Chinese Medicine, Clinical Research Team of Prevention and Treatment of Cardiac Emergencies with Traditional Chinese Medicine, Guangzhou, China; ^3^ Emergency Department of Guangdong Provincial Hospital of Traditional Chinese Medicine, Guangzhou, China

**Keywords:** coronary heart disease, percutaneous coronary intervention, Naoxintong capsule, efficacy, safety, meta-analysis

## Abstract

**Aims:**

To systematically evaluate the comprehensive effect of combining Naoxintong capsule (NXT) with Western medicine (WM) on coronary heart disease post-percutaneous coronary intervention (PCI).

**Methods:**

Randomized controlled trials (RCTs) of NXT for patients with CHD after PCI were systematically searched across multiple databases, including the Cochrane Library, PubMed, Embase, Chinese National Knowledge Infrastructure (CNKI), Chinese Science and Technology Journal Database (VIP), and Wan Fang, from inception until 31 January 2023. Study selection, data extraction, and quality assessment were performed by two independent reviewers. The quality of the included studies was evaluated using version 2 of the Cochrane risk-of-bias tool (RoB 2), and data analysis was performed using R4.2.2.

**Results:**

Fifteen RCTs conducted between 2011 and 2022 and involving 1,551 patients were identified, with 774 and 777 patients in the experimental and control groups respectively. It was found that the NXT and WM combination was superior to the WM therapy alone in terms of the effective clinical rate (odds ratio [OR] = 4.69, 95% confidence interval [CI] = 2.13–10.30), effective rate in electrocardiogram (OR = 6.92, 95% CI = 3.44–13.92), effective rate in angina (OR = 5.90, 95% CI = 3.04–11.46), left ventricular ejection fraction (mean difference [MD] = 4.94, 95% CI = 2.89–6.99), brain natriuretic peptide (MD = −294.00, 95% CI = −584.60 to −3.39), creatine kinase-MB (MD = −7.82, 95% CI = −13.26 to −2.37), major adverse cardiovascular events (OR = 0.24, 95% CI = 0.14–0.43), maximum platelet aggregation rate (MD = −8.33, 95% CI = −11.64 to −5.01), and Chinese medicine evidence score (OR = 9.79, 95% CI = 3.57–26.85). However, there was no significant difference in cardiac troponin I level reduction (MD = −0.13, 95% CI = 0.35–0.09) or the occurrence of adverse medicine events (OR = 0.92, 95% CI = 0.41–2.05). Meta-regression and subgroup analyses indicated that NXT capsule dosage, treatment duration, and patient baseline characteristics contributed to the heterogeneity.

**Conclusion:**

A combination of NXT and WM can improve clinical outcomes in patients undergoing PCI. However, further studies are needed to confirm the reliability and safety of this combined treatment approach.

**Systematic Review Registration:**

PROSPERO, https://www.crd.york.ac.uk/PROSPERO/display_record.php?RecordID=369174, Identifier CRD42022369174.

## 1 Introduction

Coronary heart disease (CHD) often stems from abnormal lipid metabolism, leading to the deposition of intravascular lipids in the endothelium of the coronary arteries. This deposition results in luminal narrowing and obstruction, thereby causing an imbalance between coronary blood flow and myocardial demand (Fan et al., 2021). Among non-communicable diseases (NCDs), cardiovascular diseases (CVDs) are the primary contributors to the global disease burden and the leading cause of death worldwide (2020). The Global Burden of Ischemic Heart Disease report indicates that approximately 9.14 million individuals succumbed to CVDs in 2019 globally, with about 197 million people living with these diseases (Safiri et al., 2022). The most common medications for CVDs include atorvastatin tablets, isosorbide mononitrate extended-release tablets, metoprolol tablets, and aspirin tablets; however, the clinical effect is not ensured when used only as a routine medicine (Feng et al., 2021). Percutaneous coronary intervention (PCI) is also a major treatment option, and in recent years, the application of this therapy has become widespread with the rising incidence of CVD (Safiri et al., 2022). It is now considered the primary treatment method for CHD (Shen et al., 2023). While it effectively improves perfusion and eases angina symptoms in patients, PCI is accompanied by postoperative complications such as thrombosis, myocardial infarction, heart failure, cognitive dysfunction, and cranial nerve injury (Shi et al., 2019). Moreover, insufficient postoperative rehabilitation care often results in repeated coronary angiography and revascularization. This hampers patients’ return to daily activities and significantly diminishes their quality of life (Tanaka et al., 2015). Therefore, exploring potential medications that can effectively prevent and treat these complications is imperative.

The primary pharmacological agents currently used for secondary prevention after PCI include antiplatelet drugs, statins, angiotensin-converting enzyme inhibitors (ACEIs), beta-blockers, and sodium-glucose cotransporter-2 (SGLT2) inhibitors (Desta et al., 2021; Virani et al., 2023). These drugs are chemically synthesized and are not derived from natural plants; hence, they are often referred to as Western medicine (WM). However, these WM treatments have certain drawbacks. For instance, antiplatelet drugs may cause severe bleeding and gastrointestinal adverse effects (Fras et al., 2021; Du et al., 2023). Statins can cause side effects such as hepatotoxicity, myotoxicity, hypotension, and newly developed diabetes (Gao et al., 2022). ACEIs may lead to hyperkalemia, renal dysfunction, and hepatotoxicity (Mustafa et al., 2022). Beta-blockers are associated with bradycardia and atrioventricular blocks (Li, 2019). SGLT2 inhibitors, which have been used in recent years as one of the drugs to reduce cardiovascular risk in nondiabetic patients, have also been associated with adverse drug reactions (ADRs) such as ketoacidosis and Fanconi syndrome (Zhang and Ji, 2021). Moreover, studies have shown that despite patients’ adherence to the recommended post-PCI pharmacological interventions, there remains a significant prevalence of adverse cardiovascular events, including stent thrombosis, myocardial infarction, and repeated revascularization procedures (Madhavan et al., 2020). Therefore, developing new adjunctive medications is crucial to improve the quality of life and survival rates of individuals receiving PCI.

The Naoxintong capsule (NXT) — a Chinese patent medicine—demonstrates a range of therapeutic effects, such as blood circulation activation, lipid regulation, and blood pressure reduction (Han et al., 2019). This capsule is composed of 16 distinct traditional Chinese medicines (TCMs), including *Astragalus mongholicus* Bunge [Fabaceae, astragali radix], *Paeonia lactiflora* Pall. [Paeoniaceae, paeoniae radix rubra], *Salvia miltiorrhiza* Bunge [Lamiaceae, salviae miltiorrhizae radix et rhizoma], *Angelica sinensis* (Oliv.) Diels [Apiaceae, angelicae sinensis radix], *Conioselinum anthriscoides* ‘Chuanxiong’ [Apiaceae, chuanxiong rhizoma], *Prunus persica* (L.) Batsch [Rosaceae, persicae ramulus], *Carthamus tinctorius* L. [Asteraceae, carthami flos], *Boswellia sacra* Flück. [Burseraceae, olibanum], *Commiphora myrrha* (T. Nees) Engl. [Burseraceae, Myrrha], *Spatholobus suberectus* Dunn [Fabaceae, spatholobi caulis], *Achyranthes bidentata* Blume [Amaranthaceae, achyranthis bidentatae radix], *Neolitsea cassia* (L.) Kosterm. [Lauraceae, cinnamomi cortex], and *Morus alba* L. [Moraceae, cortex mori]. In the Traditional Chinese Medicine Systems Pharmacology Database and Analysis Platform (TCMSP, http://tcmspw.com/tcmsp.php), the screening criteria were established as Dyslipidemia (DL) ≥0.18, Obesity (OB)≥40%, Cancer Colon 2 (Caco-2) ≥−0.4, and Hyperlipidemia (HL) ≥4 (Zhang W. et al., 2020), and the botanical drug ingredients of NXT were queried. The composition is detailed in Supplementary File S1.

Previous research has indicated that NXT exhibits cardioprotective effects and may decrease the likelihood of major adverse cardiovascular events (MACEs) post-PCI (Feng et al., 2014). The combination of NXT and WM has shown potential in enhancing clinical outcomes post-PCI, reducing myocardial injury, augmenting cardiac function, and alleviating symptoms (Fu, 2012; Li and Wei, 2016). Meanwhile, a previous meta-analysis demonstrated that the NXT and WM combination can amplify the antithrombotic effect in patients with coronary artery disease post-PCI (Liu et al., 2022). However, there is a deficiency in systematic evaluation and safety analysis concerning the comprehensive efficacy of this combination therapy, including clinical rate effectiveness, MACE rate, improvement of clinical symptoms, enhancement of cardiac function, laboratory indices, and TCM symptom scores. Herein, we performed a systematic meta-analysis of existing studies and comprehensively evaluated the combined efficacy of NXT and WM therapy in CHD patients post-PCI, which may inform clinical practice and future research.

## 2 Materials and methods

This study was conducted according to the Preferred Reporting Items for Systematic Reviews and Meta-Analyses (PRISMA) Extension Statement and was registered with the International Prospective Register of Systematic Reviews (PROSPERO, https://www.crd.york.ac.uk/PROSPERO/display_record.php?RecordID=369174, registration number CRD42022369174).

### 2.1 Search strategy

Randomized controlled trials (RCTs) of NXT for patients with CHD after PCI were thoroughly searched from seven databases, including PubMed, Embase, Cochrane Library, China National Knowledge Infrastructure (CNKI), Wan Fang Database, Chinese Science and Technology Journal Database (VIP), and Chinese Biomedical Literature, from inception until 31 January 2023. The English search terms included “Naoxintong Capsule,” “Naoxintong,” and “percutaneous coronary intervention,” The detailed search strategy employed for each database can be found in Supplementary File S2.

### 2.2 Inclusion and exclusion criteria

The inclusion criteria were as follows:

(1) Study subjects: Patients who underwent PCI.
(2) Control group treatment: The control group received WM treatments, including aspirin, statins, β-blockers, and calcium channel blockers.
(3) Experimental group treatment: The experimental group received a combination of NXT intervention and the same WM treatments as the control group.
(4) Study Design: RCTs.
(5) Selected study outcomes: Studies were considered if any of the following outcomes occurred.

Primary outcomes:

1) Effective clinical rate: This was defined as the percentage of patients who showed improvement after treatment. The effective clinical rate was calculated by subtracting the number of ineffective cases from the total number of cases and dividing the result by the total number of cases. The effectiveness of the treatment was assessed based on symptoms such as chest tightness, palpitation, shortness of breath, and weakness. Significant efficacy was defined as a substantial improvement in patients’ symptoms and signs after therapy. An effective outcome was defined as an improvement in symptoms and signs following treatment, while an ineffective outcome was defined as no significant change or a worsening of symptoms and signs after treatment (Wang et al., 2021).
2) MACE rate: This included acute coronary syndrome, heart failure, and cardiovascular death.

Secondary outcomes:

1) Effective rate in electrocardiogram (ECG): This was defined as the percentage of patients who showed improved myocardial ischemia as indicated by changes in their ECG, such as the normalization of T-wave inversion.
2) Effective rate in angina: Angina symptoms disappeared, without the need for nitroglycerin as effective; the number of angina episodes and the dosage of nitroglycerin halved as effective; angina not reduced to half or aggravated as ineffective. The effective rate in angina was calculated by subtracting the number of ineffective cases from the total number of cases and dividing the result by the total number of cases (Du et al., 2018).
3) Changes in left ventricular ejection fraction (LVEF), brain natriuretic peptide (BNP), creatine kinase-MB (CK-MB), cardiac troponin I (CTnI), and maximum platelet aggregation rate before and after treatment.
4) Chinese Medicine Evidence Score: The points of TCM symptoms were calculated according to the Guidelines for Clinical Research of New Chinese Medicines (GCRNCM). Chest tightness, shortness of breath, palpitation, and chest pain were observed in the two groups and scored according to the GCRNCM as 2 (mild), 4 (moderate), and 6 (severe). A significant effect was defined as the disappearance of symptoms before treatment, and the total score was reduced by >80%; effective was defined as the relief of symptoms before treatment, and the total score was reduced by about 40%–79%; ineffective was defined as insignificant relief of symptoms before treatment, and the total score was reduced by <40% (Qin, 2020; Gong, 2021).
5) ADRs.

The exclusion criteria were as follows:

(1) the original data is incomplete.
(2) Patients exhibit severe conditions post-PCI, including heart and renal failure.
(3) Studies exhibited either failed randomization or significant disparities in baseline data across groups.
(4) The experimental or control group received treatment with other TCMs or herbal medicines.

### 2.3 Data extraction

A blend of software and manual methods were utilized to pinpoint pertinent studies. First, all duplicate studies were removed. Next, the initial screening of titles and abstracts was independently conducted by two researchers, adhering to pre-set inclusion and exclusion criteria. A secondary screening and data extraction were then conducted by thoroughly reviewing the full text of the selected papers. The data extracted from eligible studies included the first author, year of publication, sample size, age, disease progression, treatment duration, and dosage. It also covered outcome indicators and relevant information for assessing the quality of the literature. The results were then cross-checked to ensure accuracy. Any disagreements were resolved through consensus discussions between the two reviewers or with a third party.

### 2.4 Analysis of study quality

The bias risk for the RCTs included in this meta-analysis was assessed using the Cochrane recommended risk-of-bias tool, Version 2 (RoB 2) (Sterne et al., 2019). This tool evaluated five crucial elements: the randomization process, deviations from intended interventions, missing outcome data, measurement of the outcome, and the selection of the reported result. Each element was categorized as either “low risk,” “high risk,” or “some concerns” of bias. A trial was deemed to have an overall “low risk” of bias only if all elements were classified as “low risk.” Two reviewers worked together to address and resolve disagreements to ensure accuracy and consistency. If a consensus was not achieved, a third reviewer was consulted.

### 2.5 Data analysis

All statistical analyses were performed using R statistical software (version 4.2.2) and its Meta package (version 6.5.0). Continuous and categorical variables were evaluated using the mean difference (MD) and odds ratio (OR), respectively, along with a 95% confidence interval (95% CI). The statistical significance was set at *p* < 0.05. Heterogeneity within each study was assessed using the Q statistic and I^2^ test. Forest and Labbé plots were employed to visually inspect heterogeneity and identify variation sources. The I^2^ test was also applied to evaluate heterogeneity during data integration. When I^2^ was less than 50%, indicating low heterogeneity, a fixed-effects model was applied. Conversely, a random-effects model was applied when I^2^ exceeded 50%, suggesting high heterogeneity. To address potential heterogeneity and ensure the robustness of the results, meta-regression and sensitivity analyses were performed using the “metareg” and “metainf” commands for all outcome indicators with I^2^ values ≥ 50%. In addition, subgroup analyses were conducted based on positive covariates identified in the meta-regression. Sensitivity analyses were performed by excluding each literature individually. For outcomes involving more than five studies, potential publication bias was explored using adjusted funnel plots and Egger’s and Begg’s tests, implemented via the “metabias” command.

## 3 Results

### 3.1 Literature retrieval and study characteristics

**FIGURE 1:**
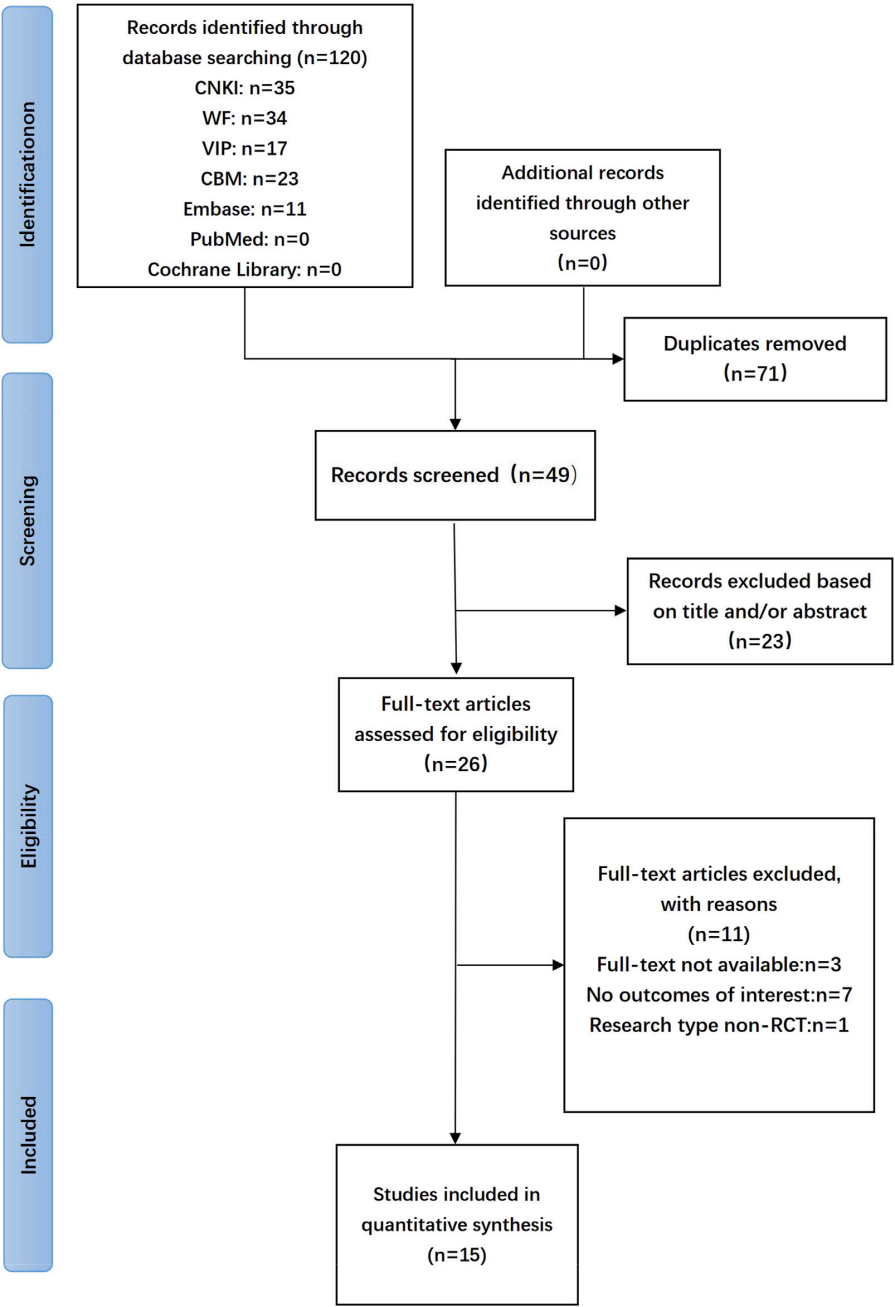
Flow diagram of systematic literature search.

A total of 120 studies were initially retrieved, of which 15 articles (He et al., 2010; Jiang and Wei, 2011; Fu, 2012; Hong, 2012; Zheng et al., 2012; Feng et al., 2014; Shen, 2014; Tian and Yang, 2015; Li and Wei, 2016; Wang and Wang, 2016; Zhang and Wang, 2017; Du et al., 2018; Zhang, 2019; Zhang Q. Y. et al., 2020; Wang et al., 2021) were included in the final analysis. The flowchart of the literature search and screening process is shown in Figure 1. The main characteristics of eligible studies are detailed in Table 1.

**TABLE 1:** Characteristics of the included studies.

Study ID	Sample size (M/F)		Age (years)		Course of disease (years)		Interventions		
	T	C	T	C	T	C	T	C	
Du et al. (2018)	61 (32/29)	60 (32/28)	52.43 ± 1.27	52.45 ± 1.26	NR	NR	NXT + WM	WM	Aspirin 300 mg po, qd
									Clopidogrel 75 mg po,qd
Fu (2012)	31 (18/13)	30 (16/14)	45–75	45–74	2–20years	1–20years	NXT + WM	WM	Digoxin 0.125 mg po, qd
									Diuretics 20 mg po, qd
									Spironolactone 2 0 mg po, qd
									ACEI po, qd
									Isosorbide dinitrate ivd, qd
Li and Wei (2016)	60 (40/20)	60 (38/22)	40–80	40–80	NR	NR	NXT + WM	WM	Aspirin 300 mg po, qd
									Clopidogrel 75 mg po,qd
									Atorvastatin 20 mg po, qd
									Metoprolol 25 mg po, qd
Zhang J (2019)	46 (28/18)	46 (26/20)	51.78 ± 6.42	51.22 ± 6.59	1–46 h	1–45 h	NXT + WM	WM	Aspirin 50–10 mg po, qd
									Clopidogrel 25–75 mg po,qd
Wang et al. (2021)	46 (17/29)	46 (21/25)	54.58 ± 4.83	54.91 ± 5.16	8.16 ± 1.27 h	8.41 ± 1.35 h	NXT + WM	WM	Warfarin
									Aspirin
									Clopidogrel
									β-receptor blocker
									Calcium Antagonists
									Isosorbide dinitrate
Jiang and Wei (2011)	80 (53/27)	80 (63/17)	61.6 ± 6.8	54.1 ± 7.8	NR	NR	NXT + WM	WM	Aspirin 100 mg po, qd
									Clopidogrel 75 mg po,qd
Tian and Yang (2015)	40 (30/10)	45 (31/14)	60.5 ± 12.6	61.2 ± 10.3	NR	NR	NXT + WM	WM	Aspirin
									Clopidogrel
									β-receptor blocker
									Atorvastatin
									ACEI
He et al. (2010)	31 (19/12)	31 (18/13)	65.2	63.9	NR	NR	NXT + WM	WM	NR
Zhang and Ji (2020)	125 (72/53)	125 (70/55)	53.29 ± 9.71	52.65 ± 9.35	NR	NR	NXT + WM	WM	NR
Wang and Wang (2016)	50 (28/22)	50 (27/23)	59.27 ± 3.35	57.26 ± 3.13	NR	NR	NXT + WM	WM	NR
Zheng et al. (2012)	27 (18/9)	27 (19/8)	71.07 ± 7.09	69.48 ± 7.87	NR	NR	NXT + WM	WM	Aspirin 100 mg po, qd
									Clopidogrel 75 mg po, qd low molecular weight heparin (LMWH) H, bid
Zhang and Wang (2017)	40 (27/13)	40 (30/10)	47–75	46–73	2–25years	3–27years	NXT + WM	WM	Aspirin
									Clopidogrel
									Bisoprolol
									Atorvastatin
Hong X (2012)	80 (53/27)	80 (53/27)	60.58 ± 10.76	61.94 ± 10.99	NR	NR	NXT + WM	WM	Aspirin 100 mg po, qd
									Clopidogrel 75 mg po,qd
Feng et al. (2014)	30 (19/11)	30 (18/12)	52.1 ± 8.6	51.9 ± 8.7	6.9 ± 2.6years	7.0 ± 2.5years	NXT + WM	WM	Warfarin
									Aspirin
									Clopidogrel
									β-receptor blocker
									Calcium Antagonists
									Isosorbide dinitrate
Shen G (2014)	27 (21/6)	27 (23/4)	55–84	54–83	NR	NR	NXT + WM	WM	Aspirin 100 mg po, qd
									Clopidogrel 75 mg po,qd
									Low molecular weight heparin (LMWH) H, bid

Abbreviations: T: treatment group; C: control group; M: males; F: females; NXT: naoxintong capsule; WM: western medication; NR: not reported.

### 3.2 Analysis of study quality

**FIGURE 2:**
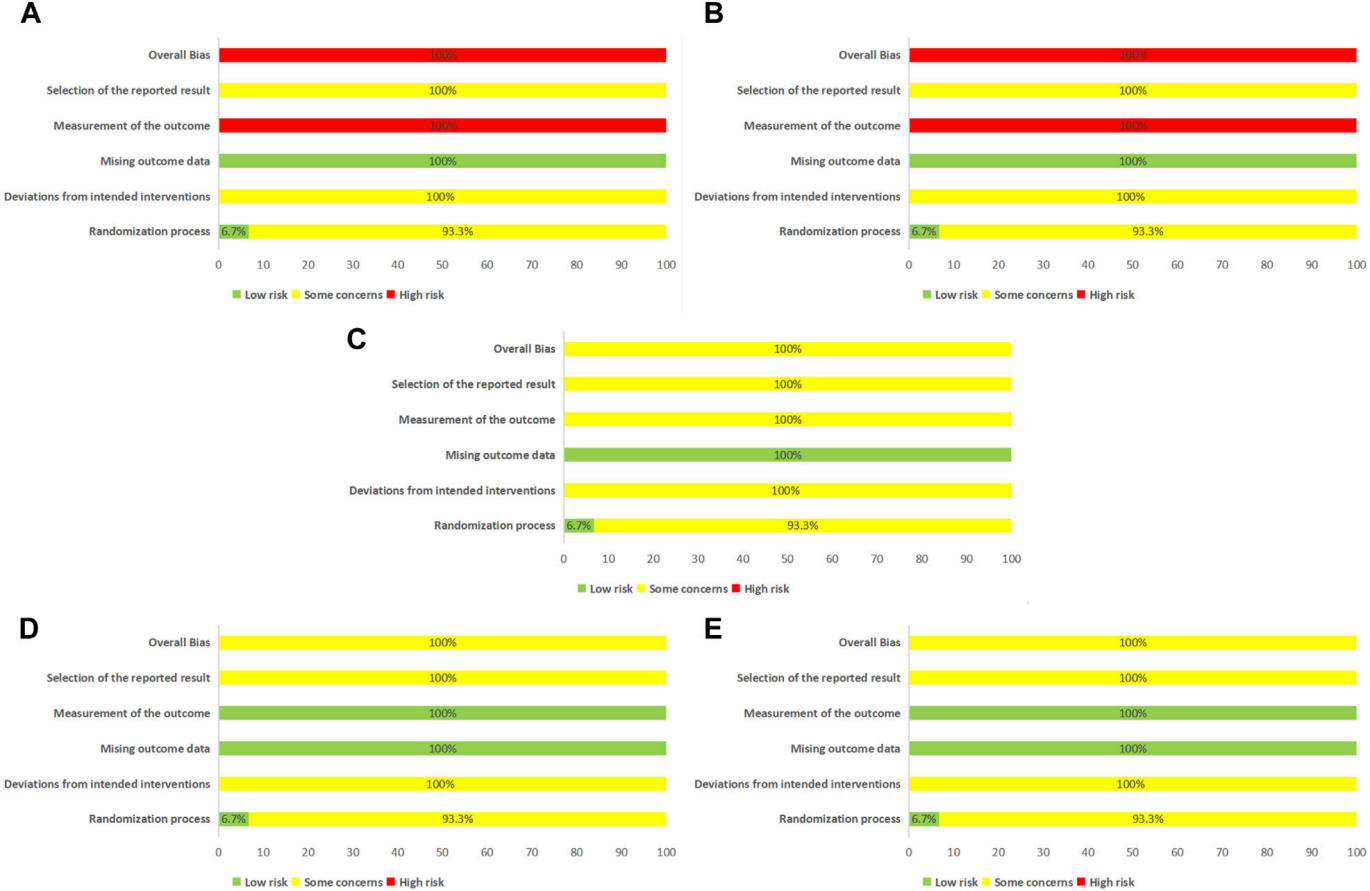
Assessment of risk bias. **(A)** Effective clinical rate; **(B)** Chinese medicine evidence score; **(C)** Effective rate in angina; **(D)** Maximum platelet aggregation rate; **(E)** CK-MB; CK-MB, Creatine Kinase-MB.

One RCT (6.67%) (Li and Wei, 2016) implemented central randomization, one RCT (6.67%) (Wang and Wang, 2016) employed parallel random sampling, one RCT (6.67%) (Zhang Q. Y. et al., 2020) utilized simple randomization through the random lottery method, and four RCTs (26.67%) (Hong, 2012; Feng et al., 2014; Zhang, 2019; Wang et al., 2021) adopted the random number table method. The remaining eight RCTs (53.33%) (He et al., 2010; Jiang and Wei, 2011; Fu, 2012; Zheng et al., 2012; Shen, 2014; Tian and Yang, 2015; Zhang and Wang, 2017) did not specify the approach used for randomization. Allocation concealment was not thoroughly explained in any of the studies, which raises “some concerns” about the randomization process. Only one RCT (6.67%) (Zheng et al., 2012) reported using a single-blind methodology, while the others did not mention blinding. None of the trials provided a clear description of the pre-designed procedure or conducted adequate analyses to assess intervention allocation effects. This led to “some concerns” about the “selection of the reported result” and “deviations from intended interventions.” Since all outcomes were evaluated based on a specific number of tested patients, there was minimal potential for bias due to missing outcome data. However, the clinical efficiency and Chinese medicine evidence scores were deemed “high risk” for “measurement of the outcome” due to their subjective nature. Based on this aspect, these studies were considered as “high risk” in “overall bias” (**Figure 2**).

### 3.3 Meta-analysis results

#### 3.3.1 Primary outcomes

##### 3.3.1.1 Effective clinical rate

**FIGURE 3:**
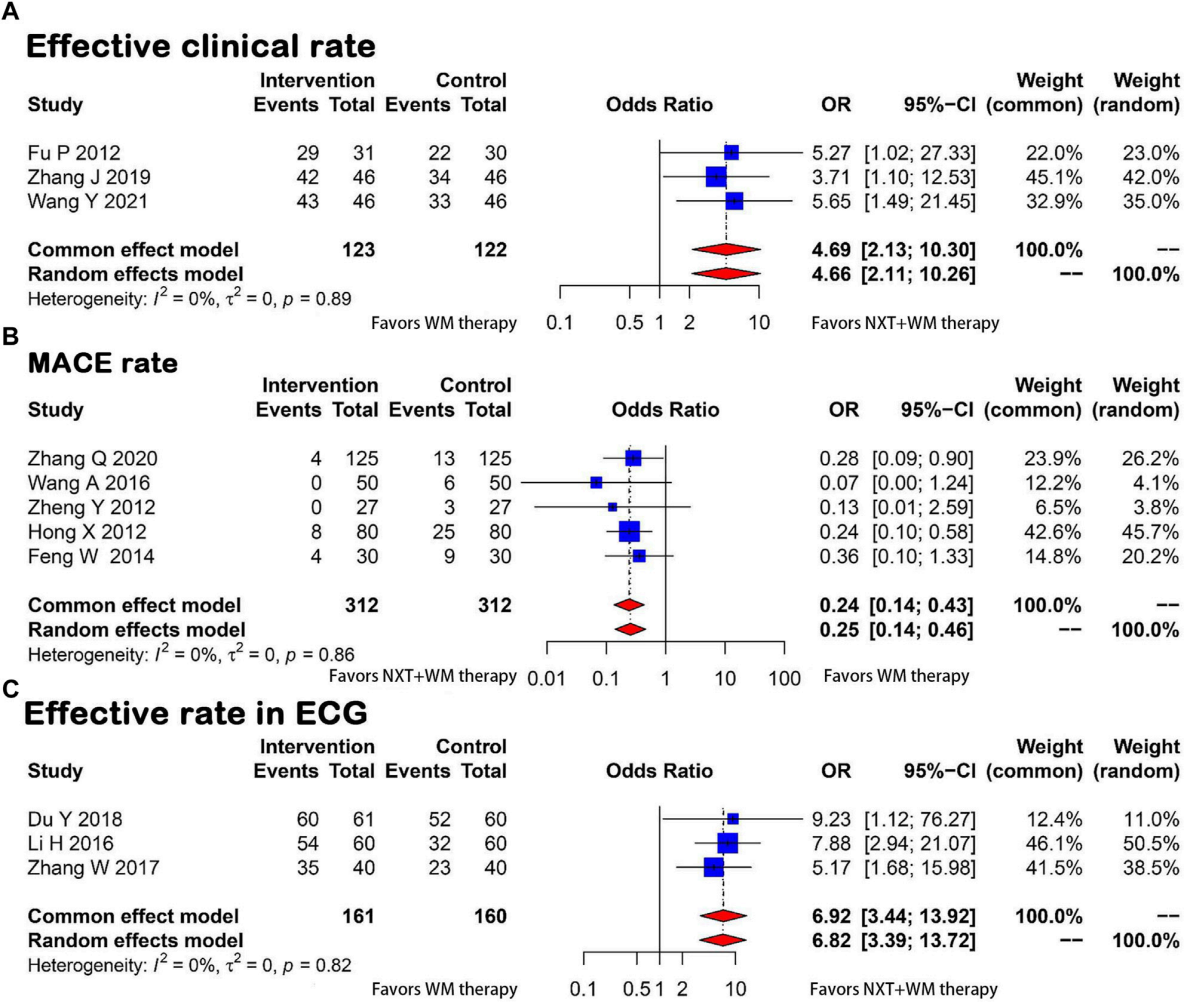
Forest plot for the meta-analysis. **(A)** Effective clinical rate; **(B)** MACE rate; **(C)** Effective rate in ECG; NXT, Naoxintong; WM, Western medicine; MACE, Major Adverse Cardiovascular Events; ECG, Electrocardiogram.

The efficacy was evaluated based on the improvement of clinical symptoms and the New York Heart Association (NYHA) classification. Three studies (Fu, 2012; Zhang, 2019; Wang et al., 2021) discussed clinical efficiency, which was analyzed using a fixed-effects model. The results showed that the intervention group had higher clinical efficiency than the control groups, and the difference was statistically significant (*p* = 0.89, I^2^ = 0, OR = 4.69, 95% CI = 2.13–10.30) (Figure 3A).

##### 3.3.1.2 MACE rate

Five studies (Hong, 2012; Zheng et al., 2012; Feng et al., 2014; Wang and Wang, 2016; Zhang Q. Y. et al., 2020) compared the incidence of MACE between the experimental and control groups. The studies were homogeneous (*p* = 0.86, I^2^ = 0), indicating that the methods and results were consistent. This was further confirmed by Labbé plots (Supplementary File S3). The results showed that the NXT and WM combination significantly reduced the incidence of MACE compared with WM therapy alone (OR = 0.24, 95% CI = 0.14–0.43) (Figure 3B).

#### 3.3.2 Secondary outcome

##### 3.3.2.1 Effective rate in ECG

Three articles (Zheng et al., 2012; Li and Wei, 2016; Du et al., 2018) reported ECG improvement rates and the heterogeneity test revealed no significant differences between groups (*p* = 0.82, I^2^ = 0), suggesting the studies were consistent. A fixed-effects model was used for analysis. The ECG improvement rate was significantly better in the test group (OR = 6.92, 95% CI = 3.44–13.92) (Figure 3C).

##### 3.3.2.2 Effective rate in angina

**FIGURE 4:**
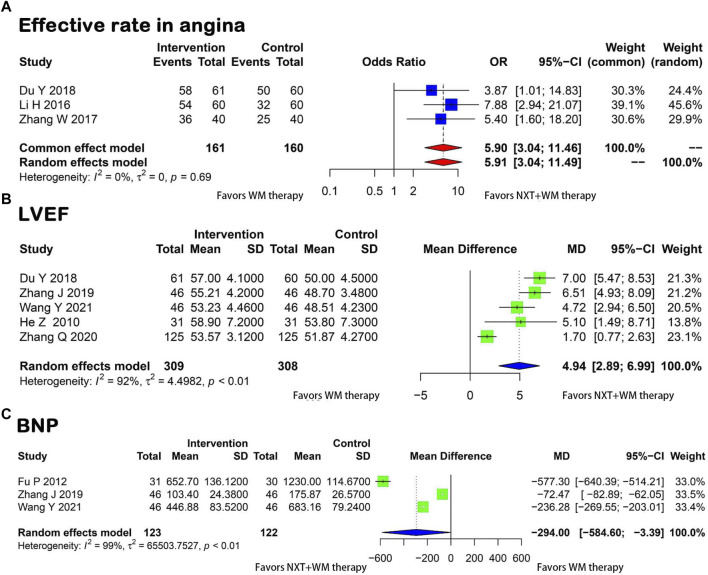
Forest plot for the meta-analysis. **(A)** Effective rate in angina; **(B)** LVEF; **(C)** BNP; NXT,Naoxintong; WM, Western medicine; LVEF, Left Ventricular Ejection Fraction; BNP, Brain Natriuretic Peptide.

Using a fixed-effects model, three articles (Zheng et al., 2012; Li and Wei, 2016; Du et al., 2018) presented the rates of improvement in angina, showing no heterogeneity across studies (*p* = 0.69, I^2^ = 0). As illustrated in Figure 4A, the meta-analysis revealed that NXT and WM combination therapy significantly improved angina symptoms compared with WM therapy alone (OR = 5.90, 95% CI = 3.04–11.46).

##### 3.3.2.3 LVEF

The analysis of five studies (He et al., 2010; Du et al., 2018; Zhang, 2019; Zhang Q. Y. et al., 2020; Wang et al., 2021) showed a high level of heterogeneity (*p* < 0.01, I^2^ = 92%) in the measurement of LVEF before and after treatment in the two groups. Due to this heterogeneity, a random-effects model was applied. The results showed a significant improvement in LVEF in the treatment group (MD = 4.94, 95% CI = 2.89–6.99) (Figure 4B).

##### 3.3.2.4 BNP

Three studies (Fu, 2012; Zhang, 2019; Wang et al., 2021) reported BNP levels in patients. A significant level of heterogeneity was found between the two groups (*p* < 0.01, I^2^ = 99%). A random-effects model was applied and results revealed a significant difference between the test and control groups, which suggested that the BNP levels decreased notably in the test group compared with the control group, suggesting that the intervention brought about an improvement in efficacy (MD = −294.00, 95% CI = −584.60 to −3.39) (Figure 4C).

##### 3.3.2.5 CK-MB

**FIGURE 5:**
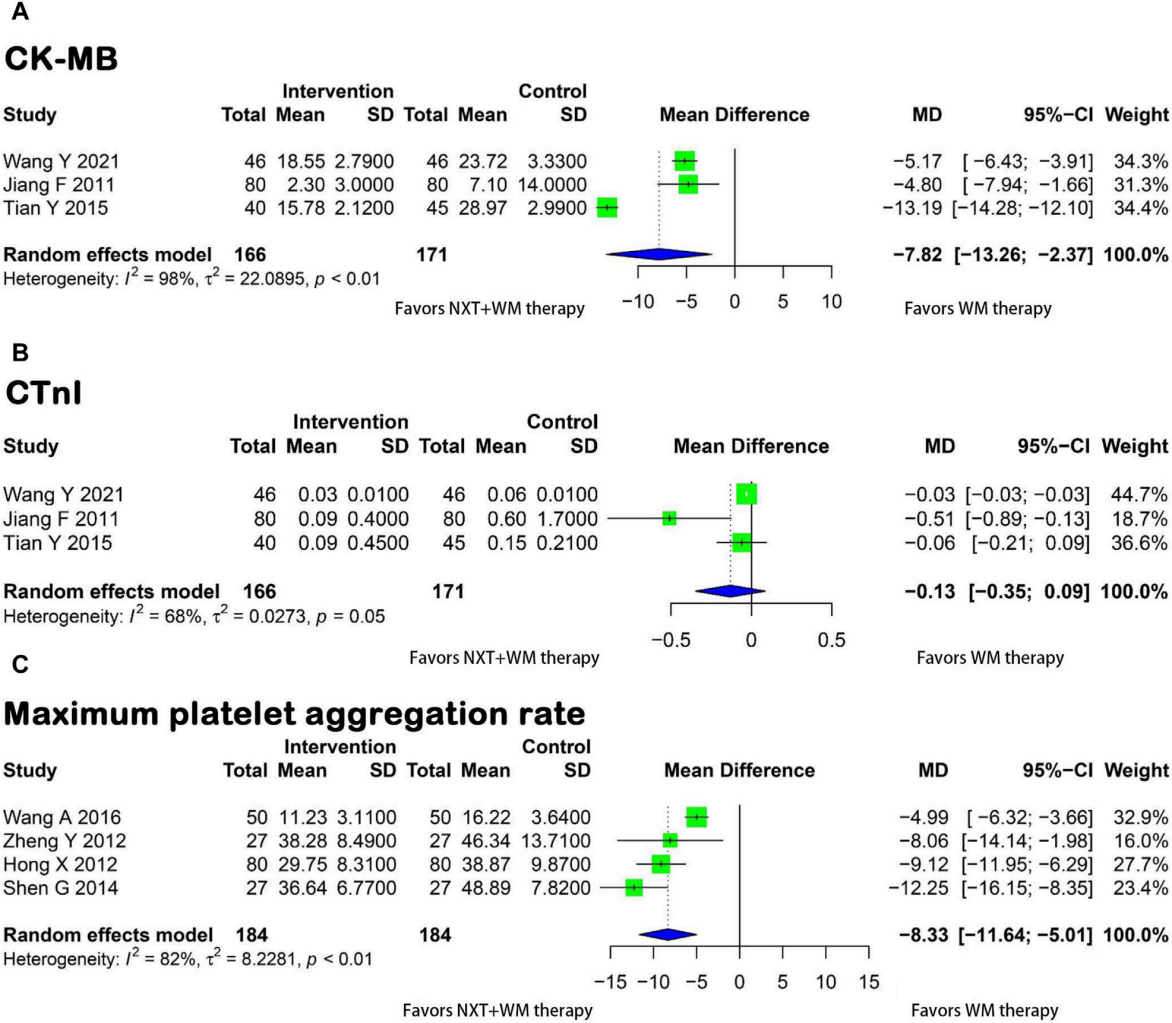
Forest plot for the meta-analysis. **(A)** CK-MB; **(B)** CTnI; **(C)** Maximum platelet aggregation rate; NXT, Naoxintong; WM, Western medicine; CK-MB, Creatine Kinase-MB; CTnI, Cardiac Troponin I

A meta-analysis of three studies (Jiang and Wei, 2011; Tian and Yang, 2015; Wang et al., 2021) showed that the experimental group had significantly lower post-treatment CK-MB levels than the control group. The difference was statistically significant (*p* < 0.01, I^2^ = 98%, MD = −7.82, 95% CI = −13.26 to −2.37) (Figure 5A).

##### 3.3.2.6 CTnI

Three studies (Jiang and Wei, 2011; Tian and Yang, 2015; Wang et al., 2021) compared CTnI levels between the experimental and control groups. However, due to heterogeneity among the trials (*p* = 0.05, I^2^ = 68%), a random-effects model was applied. The meta-analysis results showed no significant difference in the improvement of CTnI levels between the two groups (MD = −0.13, 95% CI = 0.35 to 0.09) (Figure 5B).

#### 3.3.3 Maximum platelet aggregation rate

Four studies (Hong, 2012; Zheng et al., 2012; Shen, 2014; Wang and Wang, 2016) reported the maximum platelet aggregation rate. A meta-analysis conducted using a random-effects model (*p* < 0.02, I^2^ = 82%) showed that the experimental group exhibited a lower maximum platelet aggregation rate after treatment than the control group (Figure 5C). These findings suggest that NXT significantly reduced the maximum platelet aggregation rate in patients who underwent PCI for coronary artery disease (MD = −8.33, 95% CI = −11.64 to −5.01).

#### 3.3.4 Chinese medicine evidence score

**FIGURE 6:**
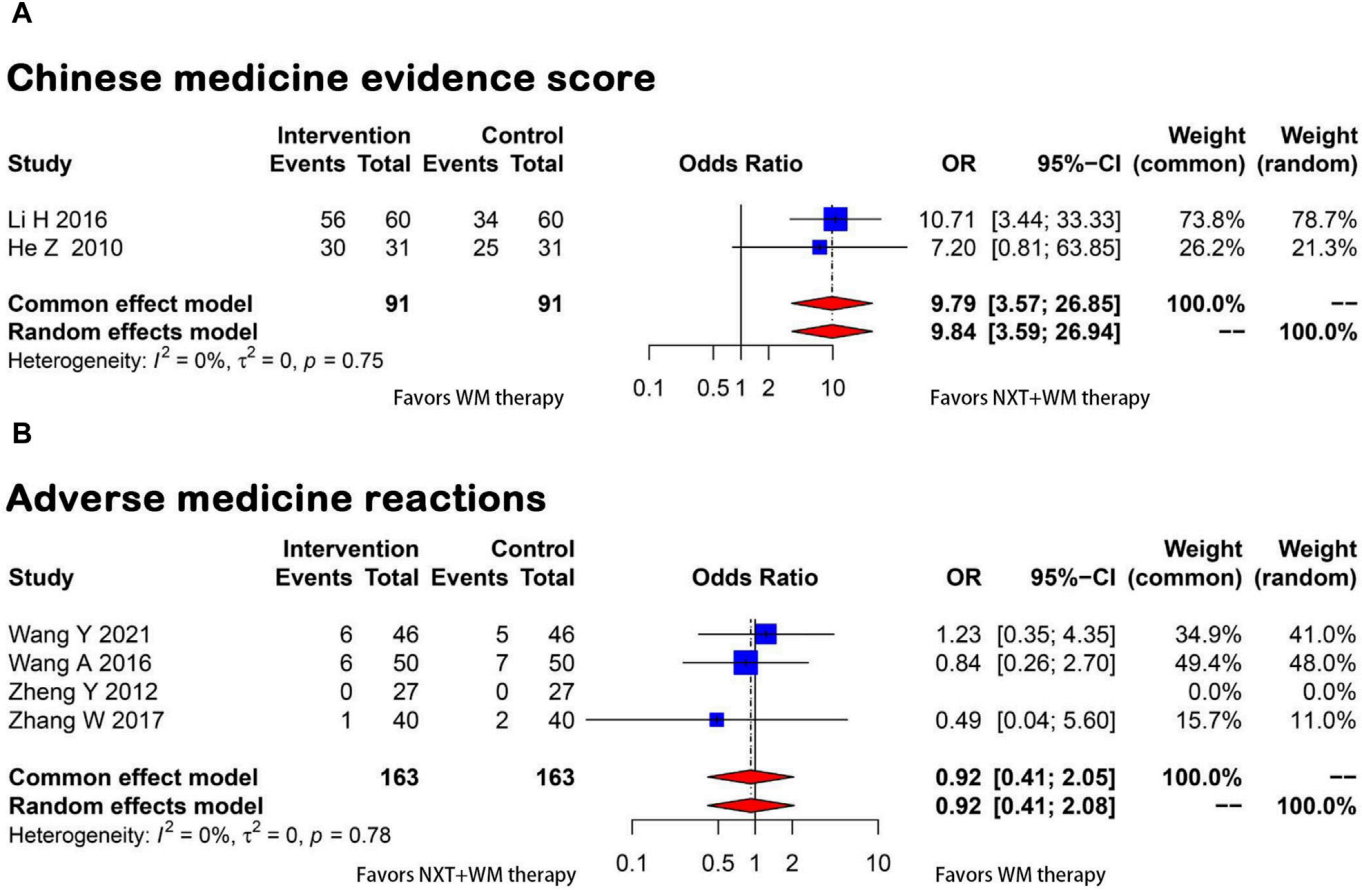
Forest plot for the meta-analysis. **(A)** Chinese medicine evidence score; **(B)** Adverse medicine reactions; NXT, Naoxintong; WM, Western medicine.

Two papers (He et al., 2010; Li and Wei, 2016) reported Chinese medicine evidence scores. Since homogeneity was found between the two studies (*p* = 0.75, I^2^ = 0), the fixed-effects model was utilized and the results demonstrated that the experimental group exhibited a significant improvement in patient’s clinical symptoms (OR = −9.79, 95% CI = 3.57–26.85) (Figure 6A).

#### 3.3.5 ADRs

Four studies (Zheng et al., 2012; Wang and Wang, 2016; Zhang and Wang, 2017; Wang et al., 2021) provided information on ADRs, such as gastrointestinal reactions and rash. A meta-analysis was conducted using a fixed-effects model, and results revealed that NXT and WM combination therapy significantly reduced the incidence of ADRs (OR = 0.92, 95% CI = 0.41–2.05). The statistical analysis showed homogeneity (*p* = 0.78 and I^2^ = 0) (Figure 6B).

#### 3.3.6 Analysis of publication bias

Publication bias for outcomes with more than five studies was assessed. The funnel plot showed approximate symmetry, employing MACE as an exemplar, indicating no significant publication bias, which was also validated by Egger’s and Begg’s tests (Egger’s test: t = −1.62, *p* = 0.2028; Begg’s test: z = −0.49, *p* = 0.6242). A non-parametric cut and fill analysis was performed, and the subsequent funnel plot displayed a *p*-value of 0.86, further confirming the absence of publication bias. However, the limited number of included studies (<10) may have introduced bias, and thus these results should be interpreted cautiously.

#### 3.3.7 Heterogeneity, meta-regression, and subgroup analyses

Meta-regression analyses were conducted on all outcome indicators with an I^2^-value ≥50%. These analyses considered three covariates: treatment duration, medication dosage, and subject age. These covariates were selected based on the characteristics of the studies included (Supplementary File S4). There was a correlation between subject age and treatment duration with a decrease in BNP levels in both the experimental and control groups. Additionally, treatment duration was associated with an improvement in the subjects’ LVEF. However, no correlation was found between the three covariates and CTnI, CK-MB, or maximum platelet aggregation rate in the two groups. Nonetheless, whether there are significant unmeasured covariates that could account for the heterogeneity observed across different studies remains unclear. When LVEF was examined in subgroups according to treatment duration, relative heterogeneity within these subgroups diminished, indicating that treatment duration significantly contributed to study heterogeneity. However, subgroup analyses of BNP based on treatment duration and subject age were inconclusive due to an insufficient number of studies (<2) within these subgroups.

#### 3.3.8 Sensitivity analysis

Sensitivity analyses were performed by examining each study individually, excluding the original LVEF study, which included over five papers and showed significant variability. After the removal of this study (Zhang Q. Y. et al., 2020), there was a significant reduction in heterogeneity. Changes were also observed in the MD and the 95% CI (Supplementary File S5). This suggests that this study could be the source of the observed heterogeneity.

## 4 Discussion

To the best of our knowledge, this is the first meta-analysis to explore the collective efficacy of NXT and WM combination therapy in CHD patients post-PCI. The results highlighted that the NXT and WM combination significantly enhances clinical effectiveness and reduces the incidence of MACEs. This dual therapy also substantially improves LVEF—a crucial determinant of cardiac function and prognosis in patients receiving PCI. The findings also demonstrated a significant increase in LVEF in the dual therapy group compared with the WM monotherapy group. Additionally, the combined therapy markedly enhances ECG and angina improvement rates. The NXT and WM combination therapy also significantly decreases BNP, CK-MB, and the maximum platelet aggregation rate but does not significantly benefit CTnI level reduction. Furthermore, the NXT and WM combination alleviates symptoms such as chest discomfort, palpitations, breathlessness, and fatigue, thereby improving the Chinese medicine evidence score. However, subgroup analyses of BNP based on treatment duration and subject age were inconclusive due to a lack of sufficient studies (<2) within subgroups. Notably, no significant difference was found between the combined and single WM treatments in reducing ADRs (Liu et al., 2022).

According to the principles of TCM, CHD belongs to “chest paralysis” and “heart pain” (Diseases, 2021). The pathogenesis of CHD in TCM is characterized by a deficiency at the root and excess at the branch. The root deficiency mainly being a deficiency of Qi and Yin and the excess at the branch includes blood stasis, phlegm turbidity, and Qi stagnation (Xia et al., 2022). Clinical practice has shown that CHD PCI, as a traumatic surgery, can easily damage the body’s meridians, leading to a depletion of vital energy (Wang et al., 2019). Therefore, Qi deficiency and blood stasis are the main symptoms after CHD PCI. NXT is the first Chinese patent medicine that combines plant and animal medicines to treat cardiovascular and cerebrovascular diseases. In the prescription, *Astragalus* is the sovereign, mainly for replenishing Qi, while animal medicines mainly clear the meridians and expel pathogenic factors. *Angelica*, Chuanxiong, Danshen, Baishao, Frankincense, Myrrh, Safflower, and Peach Kernel mainly invigorate the blood. The mechanism of NXT in treating CHD with Qi deficiency and blood stasis mainly includes anti-atherosclerosis, reducing inflammatory response, inhibiting platelet aggregation, enhancing endothelial function, and reducing myocardial ischemia/reperfusion (I/R) injury.

The quercetin in *Astragalus*, *S*. *miltiorrhiza*, and Safflower is the main active ingredient of NXT. It plays a role in anti-atherosclerosis by acting on signaling pathways such as tumor necrosis factor (TNF), interleukin-17 (IL-17), hypoxia-inducible factor-1, and vascular endothelial growth factor (Boutet et al., 2018; Xing et al., 2018; Yang et al., 2020; Singh et al., 2021). It can improve myocardial ischemia, coronary blood flow, and lipid levels, which are essential in the prevention and treatment of CVDs. Polysaccharides, primary compounds in Astragalus, have been shown to enhance macrophage capacity for oxidized low-density lipoprotein phagocytosis. This process promotes cholesterol efflux, thereby exerting anti-atherosclerosis effects (Duan et al., 2020; Ge et al., 2022). Additionally, these polysaccharides improve endothelial function and stabilize plaques by inducing macrophages to produce nitric oxide, nitric oxide synthase, and TNF-α via nuclear factor kappa B. The underlying mechanism appears to involve the promotion of the expression of α messenger RNA (mRNA) in aortic smooth muscle 22α, while simultaneously inhibiting the expression of matrix metalloproteinase-2 and TNF-α mRNA (Yang et al., 2016). Coronary artery obstruction is often caused by the activation of the coagulation system, platelet aggregation, and thrombosis. Certain substances, including hirudin, earth dragon, total paeoniflorin, *Angelica* volatile oil, and components containing ferulic acid, have been found to inhibit adenosine diphosphate-induced platelet aggregation. These substances also prolong prothrombin time (PT), activate partial thromboplastin time (APTT), and reduce blood viscosity (Martin et al., 2019; Ma et al., 2022; Wei, 2022; Li et al., 2023). Furthermore, the purified liquid from the whole scorpion has been shown to inhibit thrombosis by extending rat APTT and PT, inhibiting tissue-type plasminogen activator (tPA), and enhancing the activity of plasminogen activator inhibitor-1 (PAI-1) (Wali et al., 2019). Notably, oxidative stress reaction represents the key pathological mechanism underlying CHD, facilitating the development of atherosclerosis (D'Oria et al., 2020). Lactoperoxidase (LPO) and myeloperoxidase (MPO) serve as reliable markers for lipid peroxidation; elevated LPO levels can exacerbate myocardial injury (Chen et al., 2021), whereas MPO contributes to the generation of reactive oxygen species (Yang et al., 2023). NXT effectively inhibits adhesion molecules and delays the progression of atherosclerosis by reducing MPO and LPO levels and enhancing oxidative stress conditions (Yang et al., 2023). This may be related to our findings suggesting that NXT improves clinical efficacy and increases the rate of ECG and angina improvement.

Dyslipidemia is an independent risk factor for CHD. Studies have proposed that flavonoids and triterpenoids found in *S*. *miltiorrhiza* can potentially decrease the expression of scavenger receptor A on the surface of macrophages, ultimately inhibiting the formation of foam cells and effectively reducing blood lipid levels (Ren et al., 2019). Furthermore, pharmacological studies have substantiated that *S*. *miltiorrhiza* and Rhizoma Ligustici Chuanxiong exhibit notable protective effects against myocardial I/R injury (liu et al., 2020). It has been previously demonstrated that inflammatory responses are pivotal in precipitating cardiovascular events in patients diagnosed with CHD (Haybar et al., 2019). NXT can suppress intra-arterial inflammatory responses, thereby reducing serum levels of inflammatory markers such as TNF-α, IL-6, IL-18, monocyte chemoattractant protein 1 (MCP-1) (Luo and Liao, 2019), and metalloproteinase 9 (Lv et al., 2021). This reduction in inflammatory indicators contributes to a decrease in the incidence of adverse cardiovascular events in patients, thereby enhancing their prognosis. Our data suggest that these effects are associated with further improvements in patient prognosis, as evidenced by a reduction in MACEs.

A meta-analysis (Liu et al., 2022) evaluating the efficacy of combining NXT with WM in patients with coronary artery disease after PCI found that this combination therapy inhibited platelet activation and aggregation in patients with coronary artery disease after PCI, improved the body’s coagulation function, and increased the antithrombotic efficacy. However, this meta-analysis had limitations, as it focused solely on antithrombotic efficacy and safety, resulting in a relatively narrow scope of findings. Additionally, the study lacked sensitivity or meta-regression analysis and failed to systematically analyze sources of heterogeneity, reducing the reliability and validity of the results. Hence, the present study evaluated the comprehensive effect and safety of NXT and WM combination therapy based on clinical effectiveness, MACEs, LVEF, CK-MB, Chinese medicine evidence score, and adverse effects in CHD patients post-PCI. Moreover, meta-regression and sensitivity subgroup analyses were performed to identify and analyze sources of heterogeneity and ensure the robustness of the results.

Nonetheless, while our study yielded encouraging results, it also has some limitations. Firstly, all 15 RCTs included in this meta-analysis were conducted in China, with relatively small sample sizes. Some of these studies were characterized by inadequate sample sizes and unidentified risks, while others exhibited a pronounced risk of bias. These factors could potentially compromise the statistical power. Secondly, we observed significant heterogeneity in some outcome indicators such as BNP, CK-MB, and CTnI across the reviewed articles. This variability could be ascribed to differences in clinical trial methodologies and the diverse characteristics of the study participants. Finally, the evaluation criteria for the outcome indicators varied across studies, particularly the Chinese medicine evidence score, which lacks globally recognized outcome indicators. Thus, the current findings should be interpreted cautiously. We believe that future large-scale RCTs with standardized intervention protocols would contribute to more reliable evidence. Additionally, ensuring methodological rigor such as proper blinding and allocation concealment and the use of validated outcome measures would also enhance the quality of future studies.

## 5 Conclusion

In conclusion, the NXT and WM combination therapy can improve clinical efficacy in CHD patients after PCI. However, more high-quality studies are needed to provide more reliable evidence for clinical application.

## Data Availability

The original contributions presented in the study are included in the article/Supplementary Material, further inquiries can be directed to the corresponding authors. The authors followed the Preferred Reporting Items for Systematic Reviews and Meta-Analyses (PRISMA) Extension Statement to perform this meta-analysis, with a PRISMA checklist detailed in Supplementary Table S2.

## Supplementary material

Table S1 Supplementary File S1. Table of the main components; Supplementary File S2. Search strategy for meta-analysis; Supplementary File S3. Search strategy for meta-analysis; Supplementary File S4. Meta-Regression Analyses.

Table S2 PRISMA checklist.

Figure S1 Labbé plots of MACE (MACE, Major Adverse Cardiovascular Events).

Figure S2 LVEF Sensitivity analysis (LVEF, Left Ventricular Ejection Fraction).
