# Supplementary material for: Comprehensive effect of Naoxintong capsule combined with Western medicine on coronary heart disease after percutaneous coronary intervention: a meta-analysis
Source: Front Pharmacol. 2024 Mar 25;15:1274000. doi: 10.3389/fphar.2024.1274000 (PMC11000174; doi:10.3389/fphar.2024.1274000)
Supplement: Supplementary file 1 [file Table1.docx]

# Supplementary File S1. （table of the main components of Naoxintong capsule）

| **English/Latin Name** | **Chinese Name** | **Family** | **Species** | **Molecule ID** | **Molecule** |
| --- | --- | --- | --- | --- | --- |
| Angelicae sinensis radix / Angelica sinensis (Oliv.) Diels | Danggui | Apiaceae |  | MOL000449 | Stigmasterol |
| Astragali radix /Astragalus mongholicus Bunge [Fabaceae] | Huangqi | Fabaceae | A. membranaceus | MOL000211 | Mairin |
|  |  |  |  | MOL000239 | Jaranol |
|  |  |  |  | MOL000354 | isorhamnetin |
|  |  |  |  | MOL000371 | 3,9-di-O-methylnissolin |
|  |  |  |  | MOL000380 | (6aR,11aR)-9,10-dimethoxy-6a,11a-dihydro-6H-benzofurano[3,2-c]chromen-3-ol |
|  |  |  |  | MOL000392 | formononetin |
|  |  |  |  | MOL000398 | isoflavanone |
|  |  |  |  | MOL000417 | Calycosin |
|  |  |  |  | MOL000422 | kaempferol |
|  |  |  |  | MOL000098 | quercetin |
| Persicae ramulus / Prunus persica (L.) Batsch | Taoren | Rosaceae | - | MOL001323 | Sitosterol alpha1 |
|  |  |  |  | MOL001328 | 2,3-didehydro GA70 |
|  |  |  |  | MOL001339 | GA119 |
|  |  |  |  | MOL001340 | GA120 |
|  |  |  |  | MOL001342 | GA121-isolactone |
|  |  |  |  | MOL001343 | GA122 |
|  |  |  |  | MOL001344 | GA122-isolactone |
|  |  |  |  | MOL001351 | Gibberellin A44 |
|  |  |  |  | MOL001358 | gibberellin 7 |
|  |  |  |  | MOL001371 | Populoside_qt |
| Carthami flos / Carthamus tinctorius L. | Honghua | Asteraceae | C. tinctorius | MOL000422 | kaempferol |
|  |  |  |  | MOL002695 | lignan |
|  |  |  |  | MOL000449 | Stigmasterol |
|  |  |  |  | MOL002721 | quercetagetin |
|  |  |  |  | MOL000098 | quercetin |
|  |  |  |  | MOL002680 | Flavoxanthin |
|  |  |  |  | MOL002712 | 6-Hydroxykaempferol |
| Olibanum / Boswellia sacra Flück. | Ruxiang | Burseraceae | B. carterii | MOL001215 | tirucallol |
|  |  |  |  | MOL001241 | O-acetyl-α-boswellic acid |
|  |  |  |  | MOL001263 | 3-oxo-tirucallic,acid |
|  |  |  |  | MOL001272 | incensole |
| Myrrha / Commiphora myrrha (T.Nees) Engl. | Moyao | Burseraceae | C. myrrha | MOL001006 | poriferasta-7,22E-dien-3beta-ol |
|  |  |  |  | MOL001013 | mansumbinoic acid |
|  |  |  |  | MOL001031 | epimansumbinol |
|  |  |  |  | MOL001040 | (2R)-5,7-dihydroxy-2-(4-hydroxyphenyl)chroman-4-one |
|  |  |  |  | MOL001049 | 16-hydroperoxymansumbin-13(17)-en-3β-ol |
|  |  |  |  | MOL001052 | mansumbin-13(17)-en- 3,16-dione |
|  |  |  |  | MOL001063 | 28-acetoxy-15α-hydroxymansumbinone |
|  |  |  |  | MOL001088 | 1α-acetoxy-9,19-cyclolanost-24-en-3β-o |
|  |  |  |  | MOL001095 | isofouquierone |
|  |  |  |  | MOL001126 | [(5aS,8aR,9R)-8-oxo-9-(3,4,5-trimethoxyphenyl)-5,5a,6,9-tetrahydroisobenzofurano[6,5-f][1,3]benzodioxol-8a-yl] acetate |
|  |  |  |  | MOL001131 | phellamurin_qt |
|  |  |  |  | MOL001147 | (20R)-3β-acetoxy-16β-dihydroxydammar-24-ene |
|  |  |  |  | MOL001148 | 3β- hydroxydammar-24-ene |
|  |  |  |  | MOL000449 | Stigmasterol |
|  |  |  |  | MOL000979 | 2-methoxyfuranoguaia-9-ene-8-one |
|  |  |  |  | MOL000098 | quercetin |
|  |  |  |  | MOL000988 | 4,17(20)-(cis)-pregnadiene-3,16-dione |
| Chuanxiong rhizoma /Conioselinum anthriscoides 'Chuanxiong' | Chuanxiong | Apiaceae | L. chuanxiong | MOL001494 | Mandenol |
|  |  |  |  | MOL002135 | Myricanone |
|  |  |  |  | MOL002140 | Perlolyrine |
|  |  |  |  | MOL002157 | wallichilide |
| Spatholobi caulis/ Spatholobus suberectus Dunn | Jixueteng | Fabaceae | S. suberectus | MOL000392 | formononetin |
|  |  |  |  | MOL000417 | Calycosin |
|  |  |  |  | MOL000449 | Stigmasterol |
|  |  |  |  | MOL000461 | 3,7-dihydroxy-6-methoxy-dihydroflavonol |
|  |  |  |  | MOL000468 | 8-o-Methylreyusi |
|  |  |  |  | MOL000469 | 3-Hydroxystigmast-5-en-7-one |
|  |  |  |  | MOL000471 | aloe-emodin |
|  |  |  |  | MOL000483 | (Z)-3-(4-hydroxy-3-methoxy-phenyl)-N-[2-(4-hydroxyphenyl)ethyl]acrylamide |
|  |  |  |  | MOL000497 | licochalcone a |
|  |  |  |  | MOL000501 | Consume close grain |
|  |  |  |  | MOL000502 | Cajinin |
|  |  |  |  | MOL000503 | Medicagol |
|  |  |  |  | MOL000506 | Lupinidine |
|  |  |  |  | MOL000507 | Psi-Baptigenin |
| Achyranthis bidentatae radix / Achyranthes bidentata Blume | Niuxi | Amaranthaceae | A. bidentata | MOL000098 | quercetin |
|  |  |  |  | MOL000422 | kaempferol |
|  |  |  |  | MOL000449 | Stigmasterol |
|  |  |  |  | MOL001006 | poriferasta-7,22E-dien-3beta-ol |
|  |  |  |  | MOL002897 | epiberberine |
|  |  |  |  | MOL004355 | Spinasterol |
| Cortex mori / Morus alba L. | Sangzhi | Moraceae | M. alba | MOL000422 | kaempferol |
|  |  |  |  | MOL000729 | Oxysanguinarine |
|  |  |  |  | MOL000737 | morin |
| Salviae miltiorrhizae radix et rhizoma / Salvia miltiorrhiza Bunge | Danshen | Lamiaceae | S. miltiorrhiza | MOL001659 | Poriferasterol |
|  |  |  |  | MOL002651 | Dehydrotanshinone II A |
|  |  |  |  | MOL007041 | 2-isopropyl-8-methylphenanthrene-3,4-dione |
|  |  |  |  | MOL007045 | 3α-hydroxytanshinoneⅡa |
|  |  |  |  | MOL007048 | (E)-3-[2-(3,4-dihydroxyphenyl)-7-hydroxy-benzofuran-4-yl]acrylic acid |
|  |  |  |  | MOL007050 | 2-(4-hydroxy-3-methoxyphenyl)-5-(3-hydroxypropyl)-7-methoxy-3-benzofurancarboxaldehyde |
|  |  |  |  | MOL007058 | formyltanshinone |
|  |  |  |  | MOL007068 | Przewaquinone B |
|  |  |  |  | MOL007069 | przewaquinone c |
|  |  |  |  | MOL007070 | (6S,7R)-6,7-dihydroxy-1,6-dimethyl-8,9-dihydro-7H-naphtho[8,7-g]benzofuran-10,11-dione |
|  |  |  |  | MOL007071 | przewaquinone f |
|  |  |  |  | MOL007077 | sclareol |
|  |  |  |  | MOL007079 | tanshinaldehyde |
|  |  |  |  | MOL007081 | Danshenol B |
|  |  |  |  | MOL007082 | Danshenol A |
|  |  |  |  | MOL007088 | cryptotanshinone |
|  |  |  |  | MOL007094 | danshenspiroketallactone |
|  |  |  |  | MOL007098 | deoxyneocryptotanshinone |
|  |  |  |  | MOL007101 | dihydrotanshinoneⅠ |
|  |  |  |  | MOL007108 | isocryptotanshi-none |
|  |  |  |  | MOL007111 | Isotanshinone II |
|  |  |  |  | MOL007115 | manool |
|  |  |  |  | MOL007119 | miltionone Ⅰ |
|  |  |  |  | MOL007125 | neocryptotanshinone |
|  |  |  |  | MOL007130 | prolithospermic acid |
|  |  |  |  | MOL007140 | (Z)-3-[2-[(E)-2-(3,4-dihydroxyphenyl)vinyl]-3,4-dihydroxy-phenyl]acrylic acid |
|  |  |  |  | MOL007150 | (6S)-6-hydroxy-1-methyl-6-methylol-8,9-dihydro-7H-naphtho[8,7-g]benzofuran-10,11-quinone |
|  |  |  |  | MOL007151 | Tanshindiol B |
|  |  |  |  | MOL007152 | Przewaquinone E |
|  |  |  |  | MOL007154 | tanshinone iia |
|  |  |  |  | MOL007155 | (6S)-6-(hydroxymethyl)-1,6-dimethyl-8,9-dihydro-7H-naphtho[8,7-g]benzofuran-10,11-dione |
|  |  |  |  | MOL007156 | tanshinone Ⅵ |
| Cinnamomi cortex/Neolitsea cassia (L.) Kosterm. | Guizhi | Lauraceae | C. cassia | MOL001736 | (-)-taxifolin |
|  |  |  |  | MOL004576 | taxifolin |
|  |  |  |  | MOL011169 | Peroxyergosterol |
| Paeoniae radix rubra / Paeonia lactiflora Pall. | Chishao | Paeoniaceae | P. lactiflora | MOL000449 | Stigmasterol |
|  |  |  |  | MOL001918 | paeoniflorgenone |
|  |  |  |  | MOL001925 | paeoniflorin_qt |
|  |  |  |  | MOL004355 | Spinasterol |
|  |  |  |  | MOL006992 | (2R,3R)-4-methoxyl-distylin |
|  |  |  |  | MOL006996 | 1-o-beta-d-glucopyranosylpaeonisuffrone_qt |
|  |  |  |  | MOL007005 | Albiflorin_qt |
|  |  |  |  | MOL007008 | 4-ethyl-paeoniflorin_qt |
|  |  |  |  | MOL007012 | 4-o-methyl-paeoniflorin_qt |
|  |  |  |  | MOL007016 | Paeoniflorigenone |
|  |  |  |  | MOL007022 | evofolinB |

**Note:** The complex compounds of 13 botanical drugs of NXT were obtained from TCMSP (<http://tcmspw.com/tcmsp.php>), which is the largest noncommercial TCM database worldwide. TCMSP have collected all the 499 herbs registered in Chinese pharmacopoeia (2010), with a total of 12144 chemical（<https://tcmspe.com/load_intro.php?id=40>）

# Supplementary File S2 ：Search strategy for meta-analysis.

# Search strategy of China National Knowledge Infrastructure.

| No. | Search items |
| --- | --- |
| #1 | SU %= '经皮冠状动脉' OR SU %= '介入' OR SU %= '标准球囊血管成形术' OR SU %= '冠状动脉内支架置入术' OR SU %= 'PCI' |
| #2 | SU %= '脑心通' |
| #3 | FT = '随机' |
| #4 | #1 AND #2 AND #3 |

## Search strategy of Wanfang Database.

| No. | Search items |
| --- | --- |
| #1 | 主题:(经皮冠状动脉) or 主题:(介入) or 主题:(标准球囊血管成形术) or 主题:( 冠状动脉内支架置入术) or 主题:(PCI) |
| #2 | 主题:(脑心通) |
| #3 | 全部:(随机) |
| #4 | #1 AND #2 AND #3 |

## Search strategy of Chinese Biomedical Literature Database.

| No. | Search items |
| --- | --- |
| #1 | "冠状动脉内支架置入术"[常用字段:智能] OR "经皮冠状动脉"[常用字段:智能] OR "介入"[常用字段:智能] OR "标准球囊血管成形术"[常用字段:智能] OR "PCI"[常用字段:智能] |
| #2 | "脑心通"[常用字段:智能] |
| #3 | "随机"[全部字段:智能]) |
| #4 | #1 AND #2 AND #3 |

## Search strategy of Weipu Journal Database.

| No. | Search items |
| --- | --- |
| #1 | M=(经皮冠状动脉 OR 介入 OR 标准球囊血管成形术 OR 冠状动脉内支架置入术 OR PCI) |
| #2 | M=(脑心通） |
| #3 | U=(随机) |
| #4 | #1 AND #2 AND #3 |

## Search strategy of Pubmed.

| No. | Search items |
| --- | --- |
| #1 | "percutaneous coronary intervention" [Mesh] |
| #2 | "coronary intervention percutaneous"[Title/Abstract] OR "coronary interventions percutaneous"[Title/Abstract] OR "intervention percutaneous coronary"[Title/Abstract] OR "interventions percutaneous coronary"[Title/Abstract] OR "percutaneous coronary interventions"[Title/Abstract] OR "percutaneous coronary revascularization"[Title/Abstract] OR "coronary revascularization percutaneous"[Title/Abstract] OR "coronary revascularizations percutaneous"[Title/Abstract] OR "percutaneous coronary revascularizations"[Title/Abstract] OR "revascularization percutaneous coronary"[Title/Abstract] OR "revascularizations percutaneous coronary"[Title/Abstract] |
| #3 | #1 OR #2 |
| #4 | Naoxintong[Title/Abstract] |
| #5 | "controlled clinical trial"[Publication Type] OR "randomized controlled trial"[Publication Type] OR "equivalence trial"[Publication Type] OR "pragmatic clinical trial"[Publication Type] OR "random*"[All Fields] |
| #6 | #3 AND #4 AND #5 |

## Search strategy of Embase.

| No. | Search items |
| --- | --- |
| #1 | 'percutaneous coronary intervention'/exp OR 'angioplasty, balloon, coronary':ti,ab,kw OR 'angioplasty, transluminal coronary':ti,ab,kw OR 'angioplasty, transluminal, percutaneous coronary' :ti,ab,kw OR 'coronary angioplasty':ti,ab,kw OR 'coronary angioplasty, transluminal' :ti,ab,kw OR 'coronary artery dilatation, transluminal':ti,ab,kw OR 'coronary balloon angioplasty':ti,ab,kw OR 'p.t.c.a.':ti,ab,kw OR 'percutaneous coronary transluminal angioplasty':ti,ab,kw OR 'percutaneous transluminal coronary angioplasty' :ti,ab,kw OR 'ptca':ti,ab,kw |
| #2 | naoxintong:ti,ab,kw |
| #3 | 'randomized controlled trial'/exp OR 'equivalence trial'/exp OR 'non-inferiority trial'/exp OR 'pragmatic trial'/exp OR 'superiority trial'/exp OR 'controlled clinical trial':it OR 'randomized controlled trial':it OR 'equivalence trial':it OR 'pragmatic clinical trial':it OR 'superiority trial':it OR 'non-inferiority trial':it OR random* |
| #4 | #1 AND #2 AND #3 |

## Search strategy of Cochrane Library.

| No. | Search items |
| --- | --- |
| #1 | MeSH descriptor: [Percutaneous Coronary Intervention] explode all trees |
| #2 | (Intervention, Percutaneous Coronary; or Percutaneous Coronary Revascularization; or Coronary Intervention, Percutaneous; or Coronary Revascularization, Percutaneous; or Revascularization, Percutaneous Coronary; orRevascularizations, Percutaneous Coronary; or Coronary Interventions, Percutaneous; or Percutaneous Coronary Revascularizations; or Interventions, Percutaneous Coronary; or Percutaneous Coronary Interventions; or Coronary Revascularizations, Percutaneous):ti,ab,kw |
| #3 | #1 OR #2 |
| #4 | (Naoxintong):ti,ab,kw |
| #5 | MeSH descriptor: [Randomized Controlled Trial] explode all trees |
| #6 | (Randomized Controlled Trial):pt OR (Controlled Clinical Trial):pt OR (Equivalence Trial):pt OR (Pragmatic Clinical Trial):pt OR (random*) |
| #7 | #5 OR #6 |
| #8 | #3 AND #4 AND #7 |

# Supplementary File S3 ：Search strategy for meta-analysis.

# Search strategy of China National Knowledge Infrastructure.

| No. | Search items |
| --- | --- |
| #1 | SU %= 'Percutaneous Coronary Intervention' OR SU %= ' Intervention' OR SU %= 'Standard Balloon Angioplasty' OR SU %= 'Coronary Stent Implantation' OR SU %= 'PCI' |
| #2 | SU %= 'Naoxintong' |
| #3 | FT = 'Random' |
| #4 | #1 AND #2 AND #3 |

## Search strategy of Wanfang Database.

| No. | Search items |
| --- | --- |
| #1 | Topic:(Percutaneous Coronary Intervention) or Topic:( Intervention) or Topic:(Standard Balloon Angioplasty) or Topic:( Coronary Stent Implantation) or Topic:(PCI) |
| #2 | Topic:(Naoxintong) |
| #3 | All:(Random) |
| #4 | #1 AND #2 AND #3 |

## Search strategy of Chinese Biomedical Literature Database.

| No. | Search items |
| --- | --- |
| #1 | "Coronary Stent Implantation"[Common field: Intelligence] OR "Percutaneous Coronary Intervention"[Common field: Intelligence] OR " Intervention"[Common field: Intelligence] OR "Standard Balloon Angioplasty"[Common field: Intelligence] OR "PCI"[Common field: Intelligence] |
| #2 | "Naoxintong"[Common field: Intelligence] |
| #3 | "Random"[All fields: Intelligence]) |
| #4 | #1 AND #2 AND #3 |

## Search strategy of Weipu Journal Database.

| No. | Search items |
| --- | --- |
| #1 | M=(Percutaneous Coronary Intervention OR Intervention OR Standard Balloon Angioplasty OR Coronary Stent Implantation OR PCI) |
| #2 | M=(Naoxintong） |
| #3 | U=(Random) |
| #4 | #1 AND #2 AND #3 |

## Search strategy of Pubmed.

| No. | Search items |
| --- | --- |
| #1 | "percutaneous coronary intervention" [Mesh] |
| #2 | "coronary intervention percutaneous"[Title/Abstract] OR "coronary interventions percutaneous"[Title/Abstract] OR "intervention percutaneous coronary"[Title/Abstract] OR "interventions percutaneous coronary"[Title/Abstract] OR "percutaneous coronary interventions"[Title/Abstract] OR "percutaneous coronary revascularization"[Title/Abstract] OR "coronary revascularization percutaneous"[Title/Abstract] OR "coronary revascularizations percutaneous"[Title/Abstract] OR "percutaneous coronary revascularizations"[Title/Abstract] OR "revascularization percutaneous coronary"[Title/Abstract] OR "revascularizations percutaneous coronary"[Title/Abstract] |
| #3 | #1 OR #2 |
| #4 | Naoxintong[Title/Abstract] |
| #5 | "controlled clinical trial"[Publication Type] OR "randomized controlled trial"[Publication Type] OR "equivalence trial"[Publication Type] OR "pragmatic clinical trial"[Publication Type] OR "random*"[All Fields] |
| #6 | #3 AND #4 AND #5 |

## Search strategy of Embase.

| No. | Search items |
| --- | --- |
| #1 | 'percutaneous coronary intervention'/exp OR 'angioplasty, balloon, coronary':ti,ab,kw OR 'angioplasty, transluminal coronary':ti,ab,kw OR 'angioplasty, transluminal, percutaneous coronary' :ti,ab,kw OR 'coronary angioplasty':ti,ab,kw OR 'coronary angioplasty, transluminal' :ti,ab,kw OR 'coronary artery dilatation, transluminal':ti,ab,kw OR 'coronary balloon angioplasty':ti,ab,kw OR 'p.t.c.a.':ti,ab,kw OR 'percutaneous coronary transluminal angioplasty':ti,ab,kw OR 'percutaneous transluminal coronary angioplasty' :ti,ab,kw OR 'ptca':ti,ab,kw |
| #2 | naoxintong:ti,ab,kw |
| #3 | 'randomized controlled trial'/exp OR 'equivalence trial'/exp OR 'non-inferiority trial'/exp OR 'pragmatic trial'/exp OR 'superiority trial'/exp OR 'controlled clinical trial':it OR 'randomized controlled trial':it OR 'equivalence trial':it OR 'pragmatic clinical trial':it OR 'superiority trial':it OR 'non-inferiority trial':it OR random* |
| #4 | #1 AND #2 AND #3 |

## Search strategy of Cochrane Library.

| No. | Search items |
| --- | --- |
| #1 | MeSH descriptor: [Percutaneous Coronary Intervention] explode all trees |
| #2 | (Intervention, Percutaneous Coronary; or Percutaneous Coronary Revascularization; or Coronary Intervention, Percutaneous; or Coronary Revascularization, Percutaneous; or Revascularization, Percutaneous Coronary; orRevascularizations, Percutaneous Coronary; or Coronary Interventions, Percutaneous; or Percutaneous Coronary Revascularizations; or Interventions, Percutaneous Coronary; or Percutaneous Coronary Interventions; or Coronary Revascularizations, Percutaneous):ti,ab,kw |
| #3 | #1 OR #2 |
| #4 | (Naoxintong):ti,ab,kw |
| #5 | MeSH descriptor: [Randomized Controlled Trial] explode all trees |
| #6 | (Randomized Controlled Trial):pt OR (Controlled Clinical Trial):pt OR (Equivalence Trial):pt OR (Pragmatic Clinical Trial):pt OR (random*) |
| #7 | #5 OR #6 |
| #8 | #3 AND #4 AND #7 |

# Supplementary File S4.Meta-Regression Analyses

| **BNP** |  | estimate | se | zval | pval | ci.lb | ci.ub |
| --- | --- | --- | --- | --- | --- | --- | --- |
|  | intrcpt | 26.5407 | 1615.0337 | 0.0164 | 0.9869 | -3138.8671 | 3191.9485 |
|  | Dose | -87.6069 | 436.9153 | -0.2005 | 0.8411 | -943.9452 | 768.7314 |
|  | intrcpt | -153.5818 | 81.9012 | -1.8752 | 0.0608 | -314.1051 | 6.9415 |
|  | Age≥55 | -423.7182 | 144.9222 | -2.9238 | 0.0035 | -707.7606 | -139.6758 |
|  | intrcpt | -577.3 | 119.5603 | -4.8285 | <.0001 | -811.6338 | -342.9662 |
|  | Time≥4weeks | 423.7182 | 144.9222 | 2.9238 | 0.0035 | 139.6758 | 707.7606 |
| LVEF | intrcpt | 10.6958 | 7.9742 | 1.3413 | 0.1798 | -4.9334 | 26.325 |
|  | Dose | -1.6049 | 2.2053 | -0.7278 | 0.4668 | -5.9273 | 2.7174 |
|  | intrcpt | 4.9278 | 1.2388 | 3.9779 | <.0001 | 2.4998 | 7.3558 |
|  | Age≥55 | 0.1722 | 3.2398 | 0.0531 | 0.9576 | -6.1776 | 6.522 |
|  | intrcpt | 6.0534 | 0.5948 | 10.1769 | <.0001 | 4.8876 | 7.2192 |
|  | Time NR | -4.3534 | 1.029 | -4.2309 | <.0001 | -6.3701 | -2.3367 |
| CTn1 | intrcpt | -0.7518 | 0.5432 | -1.3839 | 0.1664 | -1.8165 | 0.313 |
|  | Dose | 0.1935 | 0.1675 | 1.1557 | 0.2478 | -0.1347 | 0.5218 |
|  | intrcpt | -0.03 | 0.2814 | -0.1066 | 0.9151 | -0.5815 | 0.5215 |
|  | Age≥55 | -0.2194 | 0.3585 | -0.6119 | 0.5406 | -0.922 | 0.4833 |
|  | intrcpt | -0.2494 | 0.2222 | -1.1224 | 0.2617 | -0.6848 | 0.1861 |
|  | Time≥4weeks | 0.2194 | 0.3585 | 0.6119 | 0.5406 | -0.4833 | 0.922 |
| CKMB | intrcpt | 5.3959 | 7.2323 | 0.7461 | 0.4556 | -8.7791 | 19.571 |
|  | Dose | -4.3422 | 2.2798 | -1.9046 | 0.0568 | -8.8104 | 0.1261 |
|  | intrcpt | -5.17 | 5.8455 | -0.8844 | 0.3765 | -16.6269 | 6.2869 |
|  | Age≥55 | -3.9592 | 7.1937 | -0.5504 | 0.5821 | -18.0586 | 10.1403 |
|  | intrcpt | -9.1292 | 4.1929 | -2.1773 | 0.0295 | -17.347 | -0.9113 |
|  | Time≥4weeks | 3.9592 | 7.1937 | 0.5504 | 0.5821 | -10.1403 | 18.0586 |
| Maximum platelet aggregation rate. | intrcpt | factors ＜ 2 levels | | | | | |
|  | Dose | factors ＜ 2 levels | | | | | |
|  | intrcpt | factors ＜ 2 levels | | | | | |
|  | Age≥55 | factors ＜ 2 levels | | | | | |
|  | intrcpt | -8.1909 | 2.7031 | -3.0302 | 0.0024 | -13.4889 | -2.8929 |
|  | Time≥4weeks | -0.5075 | 4.0192 | -0.1263 | 0.8995 | -8.385 | 7.3701 |
