# Supplementary figures and images for: Comprehensive effect of Naoxintong capsule combined with Western medicine on coronary heart disease after percutaneous coronary intervention: a meta-analysis
Source: Front Pharmacol. 2024 Mar 25;15:1274000. doi: 10.3389/fphar.2024.1274000 (PMC11000174; doi:10.3389/fphar.2024.1274000)

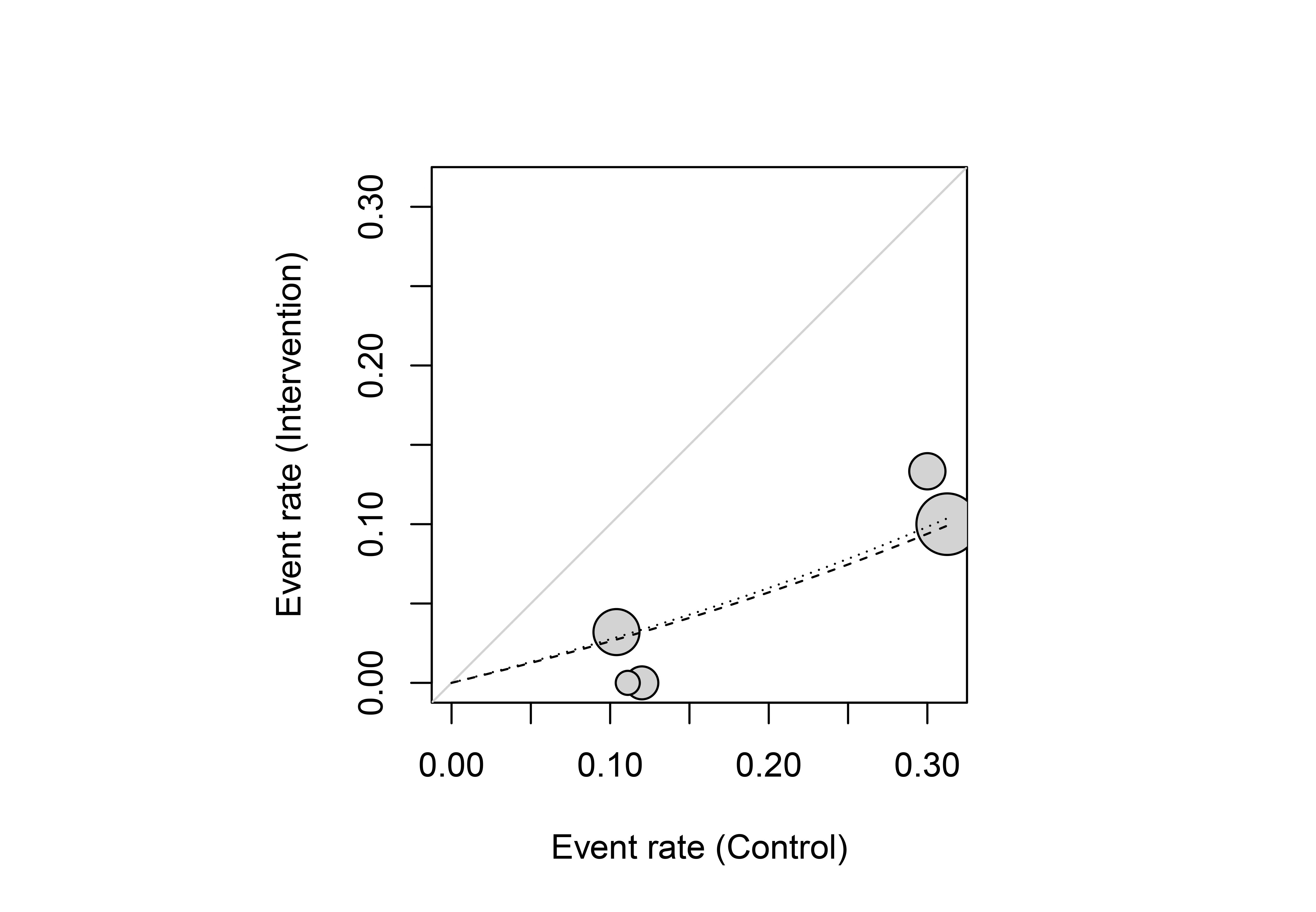

Supplement: Supplementary file 2 [file Image1.JPEG]

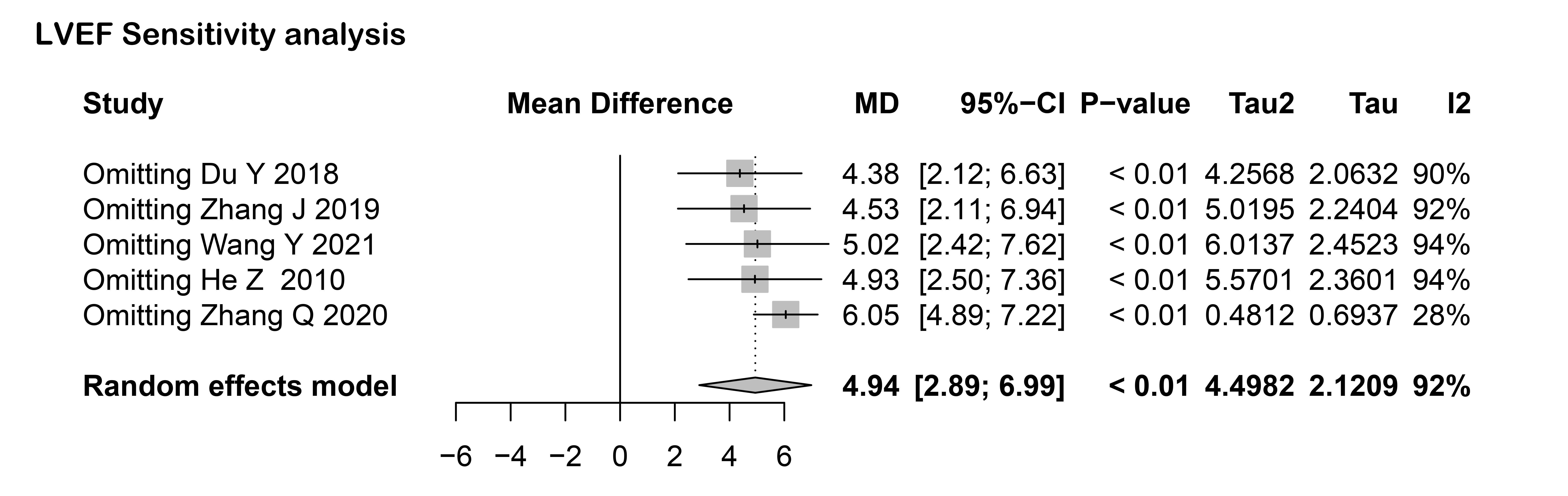

Supplement: Supplementary file 3 [file Image2.JPEG]
